# Development of eruptive keratoacanthoma-like squamous atypia after biologic therapy

**DOI:** 10.1016/j.jdcr.2025.04.034

**Published:** 2025-05-21

**Authors:** Stephen Li, Ellen Wu, Angelica Arzuaga, Michael Lewitt, Joaquin Brieva, Ahmad Amin

**Affiliations:** a Department of Dermatology, Northwestern Feinberg School of Medicine, Chicago, Illinois; b Illinois Dermatology Institute and Denova Research, Chicago, Illinois; c Chicago Medical School at Rosalind Franklin University of Medicine and Science, North Chicago, Illinois

**Keywords:** dupilumab side-effect, eruptive keratoacanthoma, eruptive squamous atypia, generalized eruptive keratoacanthoma, keratoacanthoma, tildrakizumab side-effect

## Introduction

Eruptive keratoacanthomas (KAs) are reactive squamous proliferations characterized by the appearance of scaly and often crateriform papulonodules demonstrating well-differentiated, atypical keratinocytes on histopathology. They can be found in several conditions, including familial syndromes such as Ferguson-Smith syndrome, familial KAs of Witen and Zak, and generalized eruptive KAs of Grzybowski.^1^ Treatment-induced reactive KAs have been reported in the setting of proinflammatory medications such as imiquimod,^2^ 5-fluorouracil,^3^ and immune checkpoint inhibitors (ICIs).^2^^,^^4^ However, little has been reported about their development with newer immunomodulatory biologics. We describe 2 cases of eruptive KA-like squamous atypia, secondary to dupilumab and tildrakizumab.

## Case report 1

A 63-year-old man with refractory Grover’s disease presented with a new rash 1 month after starting dupilumab. He reported a 5-year history of a pruritic rash on the chest and back that was biopsied and found to be consistent with Grover’s disease. Despite trials of high-potency topical steroids, followed by escalating doses of isotretinoin up to 80 mg daily over the course of 9 months, and then 3 months of phototherapy, the patient reported increased pruritus and lesion number. After stopping phototherapy, he was started on dupilumab for his pruritus with improvements in the first month. After his third dupilumab dose, however, he developed a new verrucous lesion on the left leg. Biopsy was consistent with invasive, well-to-moderately differentiated squamous cell carcinoma (SCC) with features of KA. He was treated with intralesional triamcinolone but subsequently developed multiple new lesions on the extremities and trunk over the next 2 months.

**Fig 1:**
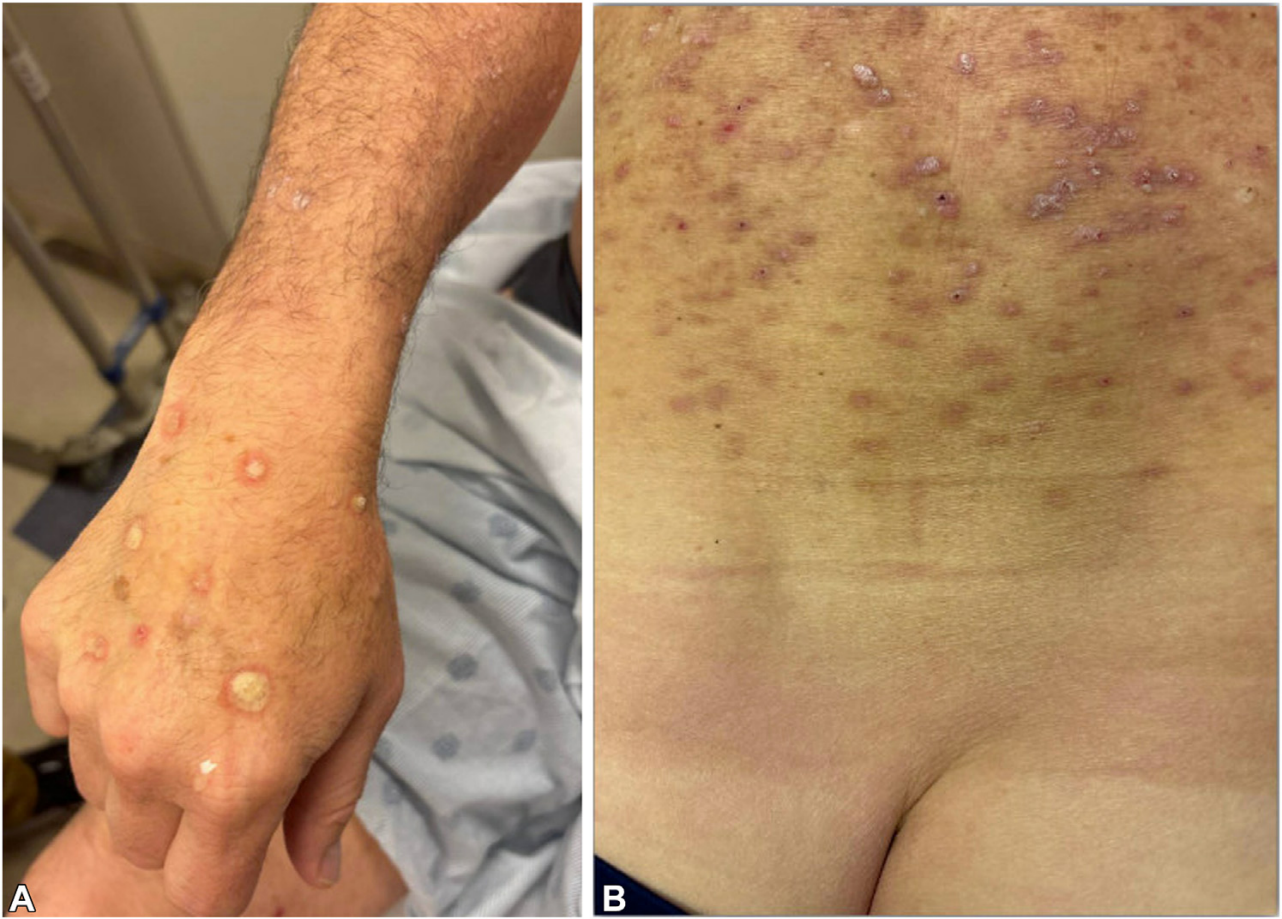
Eruptive keratoacanthanom-like squamous atypia after dupilumab. Clinical photo of the (**A**) right dorsal hand and arm, (**B**) lower back.

**Fig 2:**
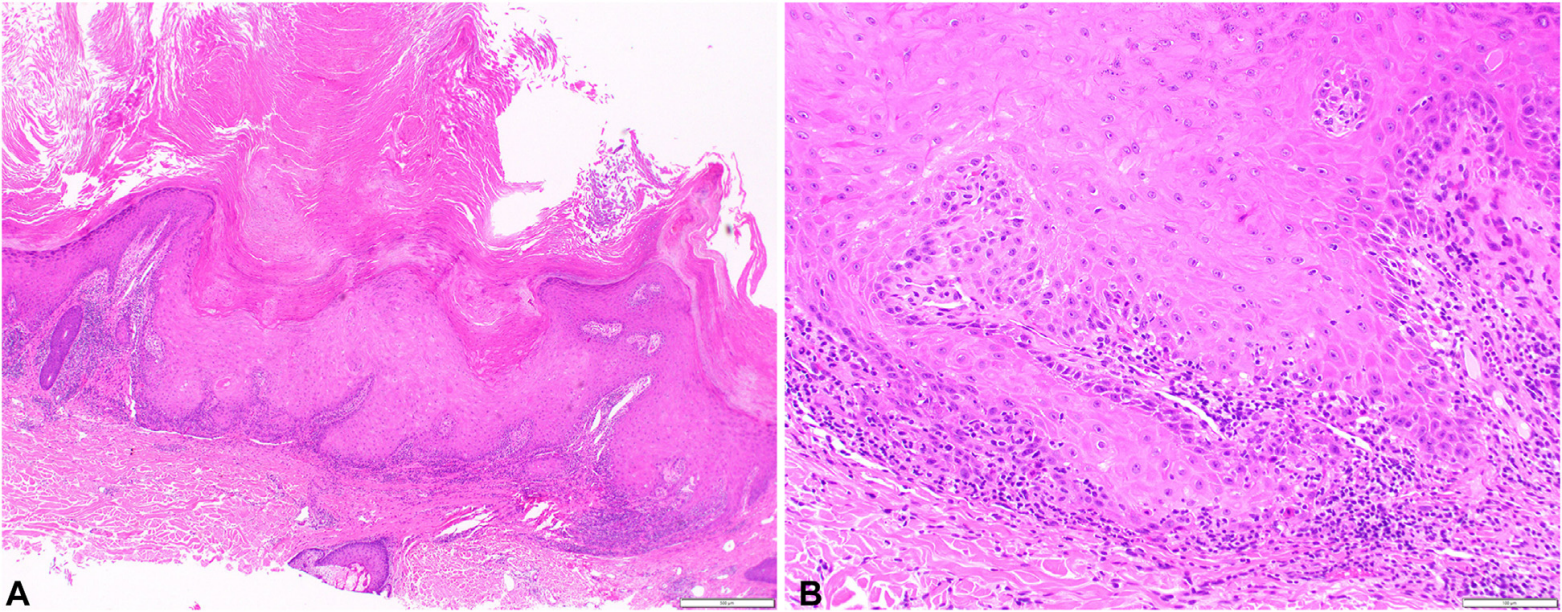
Eruptive keratoacanthanom-like squamous atypia after dupilumab histology. **A,** 4× and (**B**) 20× magnification image of hematoxylin and eosin-stained biopsy specimen from the left paraspinal back. Diagnosis was rendered as keratoacanthoma, early verrucous phase. The scale bar represents (**A**) 500 μm and (**B**) 100 μm.

On exam, the patient had hundreds of firm, pink, and keratotic papulonodules and plaques in a photodistributed pattern on the trunk and extremities (Fig 1, *A* and *B*). The patient denied a history of internal malignancy, ICI use, and personal or family history of similar rash. Review of systems was negative except for pruritic rash. Five biopsies were performed at this time with the following histologic diagnoses: Grover’s disease (chest), verruca vulgaris with superimposed features of a prurigo nodule (left flank and left calf), and consistent with KA, early verrucous phase (left paraspinal back and left lateral calf) (Fig 2, *A* and *B*). Upon clinicopathologic correlation, the patient was diagnosed with Grover’s disease with overlapping eruptive KAs in the setting of dupilumab.

After shared decision-making, dupilumab was held, and the patient was started on oral acitretin 25 mg daily and clobetasol 0.05% ointment twice daily. A trial of cryosurgery was performed on several keratotic papulonodules on the hand. The pruritus from his Grover’s remained; however, the KA lesions gradually resolved over the next few months.

## Case report 2

A 73-year-old man with a chronic history of psoriasis presented for a worsening rash 1 month after starting tildrakizumab. His psoriasis was previously managed with a variety of topical corticosteroids (most recently triamcinolone), but progression over the past year prompted concurrent initiation of tildrakizumab. A few weeks after his second (week 4) dose of tildrakizumab, the patient rapidly developed a distinct new photodistributed keratotic rash. Despite the transition to topical clobetasol 0.05% ointment, the patient’s rash continued to progress. Biopsy of a lesion 3 weeks before his third dose demonstrated prurigo nodularis.

**Fig 3:**
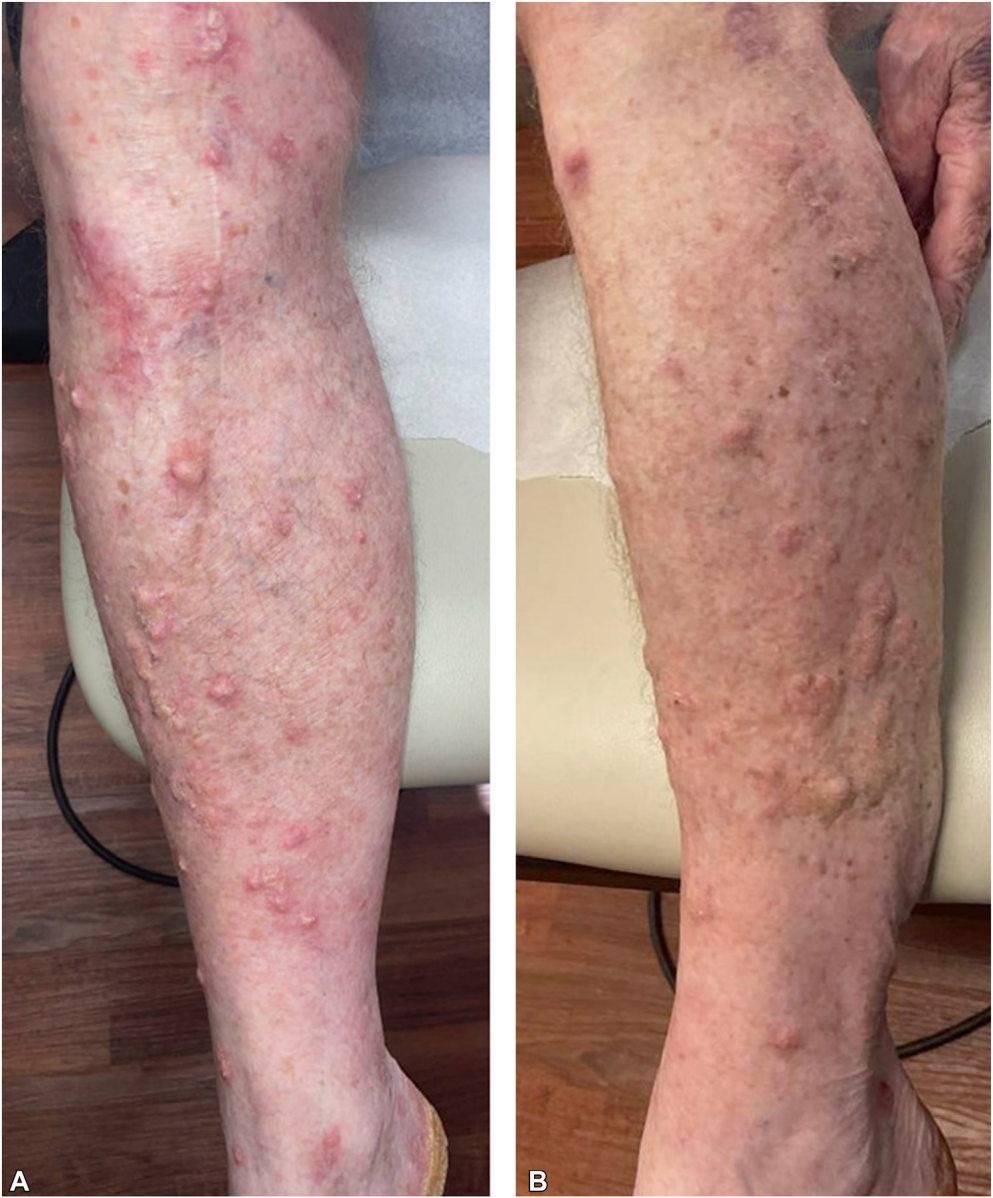
Eruptive keratoacanthanom-like squamous atypia after tildrakizumab. Clinical photos of the (**A**) right and (**B**) left legs.

**Fig 4:**
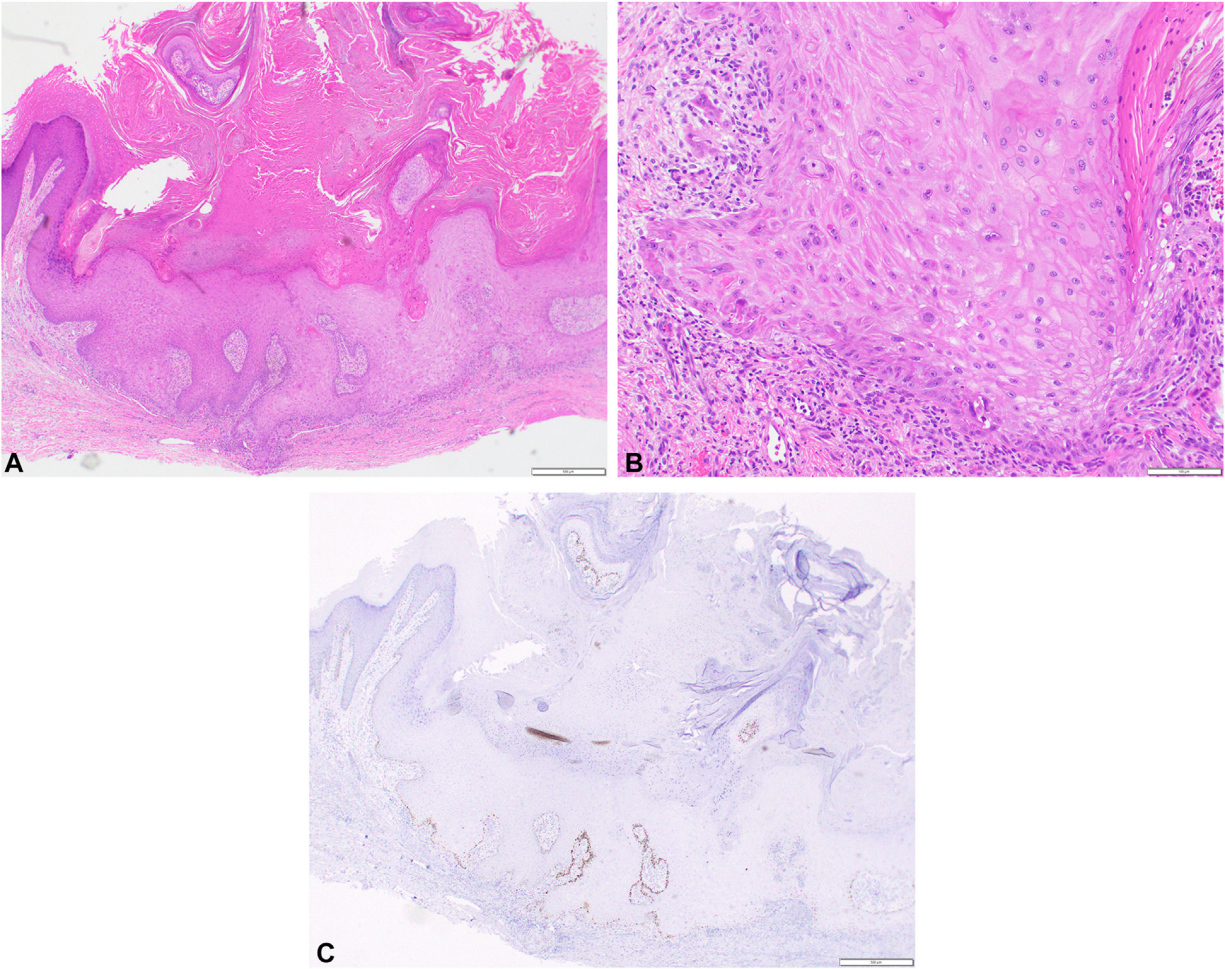
Eruptive keratoacanthanom-like squamous atypia after tildrakizumab histology. **A,** 4× and (**B**) 20× magnification images of hematoxylin and eosin-stained biopsy specimen from the right leg. **C,** Immunohistochemistry for p53 demonstrating weak positivity in the basal layer. The scale bar represents (**A** and **C**) 500 μm and (**B**) 100 μm.

On exam, the patient had numerous pink hypertrophic scaly papules and plaques favoring the bilateral legs and arms (Fig 3, *A* and *B*). The bilateral plantar feet also had erythema and mild keratoderma. The patient denied any history of internal malignancy, ICI use, and personal or family history of similar rash. Review of systems was negative except for pruritic rash. The patient denied any recent medication changes besides starting tildrakizumab. Two repeat biopsies were performed, both initially read as well-differentiated and invasive SCC (Fig 4, *A* and *B*). After discussion with dermatopathology, wild-type p53-staining was performed and found to be weakly positive in the basal layer, which can be found in reactive lesions^5^ (Fig 4, *C*). Upon clinicopathologic correlation, the diagnosis was favored to be eruptive KA-like squamous atypia in the setting of tildrakizumab.

The patient received his third (week 16) dose of tildrakizumab and started topical clobetasol 0.05% ointment under occlusion. Within 1 month, his rash resolved except for 2 remaining erythematous and centrally keratotic papulonodules on the bilateral lower legs. Biopsy of these lesions demonstrated SCC, KA growth pattern, further supporting the clinical diagnosis of eruptive KA in the setting of tildrakizumab. The patient has continued on tildrakizumab without recurrence of any lesions.

## Discussion

Eruptive KAs are reactive squamous proliferations that can often be mistaken for well-differentiated SCC. Clinicopathologic correlation is often needed to reach an accurate diagnosis. Our 2 cases demonstrate eruptive KA-like squamous atypia secondary to the immunomodulatory biologics dupilumab and tildrakizumab.

The development of KAs in relation to immunomodulatory biologics has been rarely reported. A case of dupilumab-induced eruptive KAs has previously been reported.^6^ Two cases of multiple SCCs have been reported in the context of ustekinumab (interleukin [IL]-12/23 inhibition); however, KA-like features were not noted.^7^ The mechanism of medication-induced KA formation is unknown, though proposed mechanisms largely center around the creation of a proinflammatory milieu. The development of eruptive KAs in immunosuppressed transplant and systemic lupus erythematosus patients suggest that the mechanism may be more complex.^8^ Our cases demonstrating eruptive KA-like squamous atypia secondary to blockade of IL-4/13 and IL-23 suggest that shifts in the inflammatory axis may trigger initiation of KA formation. In the case of eruptive KA secondary to ICIs, patients may experience resolution of the KAs while still continuing the offending agent.^9^ The patient in our second case also experienced resolution with topical steroids and continuation of tildrakizumab. Treatment of eruptive KAs is challenging and often multimodal. Topical options include corticosteroids, retinoids, and 5-fluorouracil. Lesion-directed therapies include surgical excision, laser therapy, and intralesional corticosteroids, bleomycin, and 5-fluorouracil.^10^ For generalized eruptive KAs, systemic treatment, particularly with oral retinoids, is largely favored.^10^

In summary, our cases add further evidence for eruptive KA-like squamous atypia potential after anti-IL-4/13 therapy and provide, to our knowledge, the first reported case of eruptive KA after anti-IL-23 therapy. Together, they further the hypothesis that nonspecific shifts in the immune microenvironment may trigger KA-like squamous atypia initiation.

## Conflicts of interest

None disclosed.
